# *Schistosoma mansoni* Infection Can Jeopardize the Duration of Protective Levels of Antibody Responses to Immunizations against Hepatitis B and Tetanus Toxoid

**DOI:** 10.1371/journal.pntd.0005180

**Published:** 2016-12-07

**Authors:** Diana K. Riner, Eric M. Ndombi, Jennifer M. Carter, Amos Omondi, Nupur Kittur, Emmy Kavere, Harrison K. Korir, Briana Flaherty, Diana Karanja, Daniel G. Colley

**Affiliations:** 1 Center for Tropical and Emerging Global Diseases and Department of Microbiology, University of Georgia, Athens, Georgia, United States of America; 2 Centre for Global Health Research, Kenya Medical Research Institute, Kisumu, Kenya; 3 Department of Pathology, Kenyatta University, Nairobi, Kenya; University of Manchester, UNITED KINGDOM

## Abstract

**Background:**

Schistosomiasis is a disease of major public health importance in sub-Saharan Africa. Immunoregulation begins early in schistosome infection and is characterized by hyporesponsiveness to parasite and bystander antigens, suggesting that a schistosome infection at the time of immunization could negatively impact the induction of protective vaccine responses. This study examined whether having a *Schistosoma mansoni* infection at the time of immunization with hepatitis B and tetanus toxoid (TT) vaccines impacts an individual’s ability to achieve and maintain protective antibody levels against hepatitis B surface antigen or TT.

**Methods:**

Adults were recruited from Kisumu Polytechnic College in Western Kenya. At enrollment, participants were screened for schistosomiasis and soil transmitted helminths (STHs) and assigned to groups based on helminth status. The vaccines were then administered and helminth infections treated a week after the first hepatitis B boost. Over an 8 month period, 3 blood specimens were obtained for the evaluation of humoral and cytokine responses to the vaccine antigens and for immunophenotyping.

**Results:**

146 individuals were available for final analysis and 26% were *S*. *mansoni* positive (Sm+). Schistosomiasis did not impede the generation of initial minimum protective antibody levels to either hepatitis B or TT vaccines. However, median hepatitis B surface antibody levels were significantly lower in the Sm+ group after the first boost and remained lower, but not significantly lower, following praziquantel (PZQ) treatment and final boost. In addition, 8 months following TT boost and 7 months following PZQ treatment, Sm+ individuals were more likely to have anti-TT antibody levels fall below levels considered optimal for long term protection. IL-5 levels in response to in vitro TT stimulation of whole blood were significantly higher in the Sm+ group at the 8 month time period as well.

**Conclusions:**

Individuals with schistosomiasis at the start the immunizations were capable of responding appropriately to the vaccines as measured by antibody responses. However, they may be at risk of a more rapid decline in antibody levels over time, suggesting that treating schistosome infections with praziquantel before immunizations could be beneficial. The timing of the treatment as well as its full impact on the maintenance of antibodies against vaccine antigens remains to be elucidated.

## Author Summary

Vaccines are a mainstay for the prevention of morbidity and mortality to numerous infectious diseases. Concurrent schistosomiasis infection at the time of immunizations has been implicated in the impairment of protective immune responses to vaccines. We asked if schistosomiasis at the initiation of the hepatitis B vaccine series and tetanus toxoid boost in adults would impact the subsequent immune responses to those vaccines. We found that *Schistosoma mansoni* infection did not block the production of antibodies to either tetanus toxoid or hepatitis B vaccine. However, the kinetics of the antibody responses differed between the schistosomiasis-infected and control groups, with lower median antibody titers to hepatitis B vaccine and a more rapid decline of antibodies against tetanus toxoid in the *S*. *mansoni*-positive group. The data indicate that this could put the individuals who are positive for *S*. *mansoni* at the start of primary or secondary immunizations at risk for losing protective antibody levels more quickly than those without schistosomiasis.

## Introduction

It is estimated that globally over 240 million people have schistosomiasis, with the bulk of cases occurring in sub-Saharan Africa [1,2]. A vast majority of those infected in the region harbor either *Schistosoma mansoni*, *S*. *haematobium* or both [3], with an estimated 122 million cases occurring in east Africa [4]. In western Kenya, near Lake Victoria where this study takes place, *S*. *mansoni* infections are common in schoolchildren. Prevalence in this population often reaches 50% or higher but decreases as distance from the lake increases [5]. There is a paucity of information on schistosomiasis prevalence levels in Kenyan adults. However, recent studies in Western Kenya suggest that prevalence in 9–12 year olds, is an excellent predictor of the prevalence in adults [6]. Thus, schistosomiasis is an ongoing community level public health problem in western Kenya. The current study is designed to determine if this situation influences standard adult immunizations in those who have or do not have *S*. *mansoni* infections at the time of their immunizations [7].

Helminths, including schistosomes, are remarkable in their ability to modulate immune responses in their host, presumably to promote their own survival. Their modulation of immune responsiveness has been shown to affect both responses to schistosome antigens and to bystander antigens [8–12]. Helminth infections have also been implicated in diminished or altered immune responses to a number of other infectious diseases including malaria [13] [14], *Helicobacter pylori* [15], HIV [16,17], and *Mycobacterium*. *tuberculosis* [18]. Also, *S*. *mansoni* and hepatitis B co-infection has been associated with more severe liver disease [19].

In murine models, harboring a helminth infection at the time of immunizations has been shown to skew immune responses to vaccine antigens against diphtheria [20], HIV [21], pneumococcus [22], and hepatitis B [23]. In human populations, diminished responses to tetanus vaccination have been reported in individuals with schistosomiasis [24], lymphatic filariasis [25], and onchocerciasis [26]. Helminth infections have also been implicated in the diminished efficacy of other established vaccines, such as Bacille Calmette-Guerin (BCG) [27–30] and cholera vaccines [31,32]. They have also been reported to lower responses to an experimental *Plasmodium falciparum* vaccine [33] and it is hypothesized that they may impede progress on the development of other experimental vaccines against malaria, HIV, and helminths [9,10], because clinical trials of these vaccine candidates often need to be done in areas where these diseases are co-endemic with helminth infections. However, in contrast to the studies cited above, a study in Gabonese children infected with *S*. *hematobium* showed no detrimental responses to tetanus toxoid (TT) boost [34] and females harboring helminth infections in Uganda and Tanzania responded as well as controls to the human papillomavirus (HPV) vaccine [35,36]. Hepatitis B vaccination in Egyptian adults infected with *S*. *mansoni* showed mixed results. When plasma derived vaccine was used, individuals with hepatosplenic schistosomiasis did not respond to the vaccine series [37] but individuals with less severe schistosomiasis did respond. However, even for those who did respond, 5 years after completion of the vaccine series, antibody responses had dropped below protective levels in 38% of those who completed follow-up and less than optimal protection levels were maintained in another 23% [38]. When recombinant hepatitis B vaccine was used in another study of Egyptian males, who were treated for their schistosomiasis after the first boost, responses were robust immediately following the completion of the series. However, no uninfected control group was included in the study for comparison nor were responses of schistosome-infected individuals followed after completion of the vaccine series [39].

The Global Vaccine Action Plan seeks to greatly increase vaccine coverage in the developing world [40]. While these efforts are primarily focused on children and pregnant women, there is a distinct need to immunize all African adults in terms of preventing infections that can cause epidemics such as meningococcal disease [41] or cholera [42] and to protect healthcare workers [43]. To evaluate the potential influence schistosomiasis may have on responses by young adults to different vaccines, we investigated primary responses to hepatitis B vaccination and secondary responses to TT in individuals who did or did not have *S*. *mansoni*. We found that individuals with schistosomiasis at the time of the initiation of the vaccinations responded to the hepatitis B vaccine series and TT boost, surpassing the minimum antibody levels associated with protection. However, the schistosomiasis positive group’s median antibody responses to hepatitis B surface antigen were generally lower following the second and third doses of the vaccine series, and for tetanus boost many of them had dropped below 1 IU/ml the level considered optimal for long term protection [44]. In light of these findings, we believe that having schistosomiasis at the time of vaccination may be detrimental to the maintenance of long term antibody responses to hepatitis B and tetanus vaccines in some young adults.

## Materials and Methods

### Study participants

Study participants were recruited from Kisumu Polytechnic College (KPC) located in Kisumu, Kenya. Study inclusion criteria were as follows: participants were required to be staff or students of KPC, be healthy men or women ≥18 years of age, and available to participate in the study for approximately 10 months. Inclusion in the study also required that participants attend a voluntary counseling and testing (VCT) clinic to ascertain their HIV status before we could evaluate their blood for the presence of antibodies to HIV. All study participants gave written informed consent prior to enrollment. Study procedures were approved by the institutional review boards of the University of Georgia (UGA), the Centers for Disease Control and Prevention (CDC), the Scientific Steering Committee of the Kenya Medical Research Institute (KEMRI), and the KEMRI/National Ethics Review Committee of Kenya. Details of the study protocol are available in the STROBE Checklist (S1 Fig).

### Laboratory methods

Participants were required to submit 3 stools on 3 consecutive days in order to ascertain helminth infection status. Stool samples were collected and processed on the same day using the Kato-Katz thick smear method [45] with 2 slides per stool for the quantitative determination of *S*. *mansoni* and the detection of the soil transmitted helminths (STH). Slides were read within an hour of preparation by 2 trained microscopists in order to detect hookworm, *Ascaris lumbricoides*, and *Trichuris trichiura* eggs. The microscopists re-examined the slides at least 24 hours after preparation to ascertain the presence of *S*. *mansoni* eggs. Results were recorded as the number of eggs per gram of stool (EPG) for *S*. *mansoni* and positive or negative for STH infections. Under the supervision of the KPC student medical clinic, individuals positive for *S*. *mansoni* eggs were treated with 40mg/kg of praziquantel. Individuals positive for hookworm, *A*. *lumbricoides*, and *T*. *trichiura* eggs were treated with 500 mg of albendazole.

### Demographic information

This study took place in an urban university setting with students potentially coming from diverse geographical regions of Kenya. As the students were living now in an urban university setting with access to piped drinking water, showers and flush toilets their exposure to *S*. *mansoni* and STH most likely occurred previously at their family homes. To assist in assessing possible previous exposure to these infections, a structured, validated questionnaire was given to participants at baseline by trained interviewers to ascertain participants’ home districts, socio-economic status (SES) and water exposure. SES was determined using 5 proxy measures including family land ownership, source of household drinking water, type of household toilet, and type of flooring and roof construction in residence. Water exposure was determined by asking about participant contact with water from lakes, ponds, rivers, or streams for work or routine daily activities. The SES and water exposure questionnaire is presented as S2 Fig.

### Vaccines administered

The vaccines used in this study were those employed in Kenya Ministry of Health vaccination programs. Hepatitis B vaccine (Elovac- B, HBI, division of Indian Immunologicals, Limited) was administered in a 3 dose series as recommended by the manufacturer at time 0, 1 month and 6 months. A small subset of individuals were unavailable to receive the final dose of hepatitis B vaccine at 6 months but were available to provide a final blood sample at 8 months. Those individuals were offered and administered the third dose at the time of the final blood draw. A booster dose of tetanus toxoid vaccine (Tetanus Vaccine (Absorbed) I.P. Serum Institute of India) was administered at time 0 as well. All vaccinations were given by a Kenya Ministry of Health nurse in conjunction with the Kenya Ministry of Health.

### Blood collection and processing

Venous blood was collected at baseline, 7 weeks, and 8 months. At each time point, thick and thin blood smears were made and slides read by well-trained microscopists to detect malaria infection. Individuals with positive malaria blood smears were referred to the KPC student medical clinic for appropriate treatment. Hemoglobin was measured at baseline and 8 months using hemocuvettes (EFK Diagnostics, Poland) and the Hemo Control reader (EFK Diagnostics, Germany). Individuals with hemoglobin levels below 8 g/dL were referred to the KPC student medical clinic for appropriate care. HIV screening was performed using the Determine HIV-1/2 test (Abbott Laboratories Abbott Park, IL) on baseline plasma samples of participants whose attendance of a VCT clinic had been confirmed. Participant plasma samples for each time point were collected and stored at -20C until being thawed and transferred using ViveST sample transport matrices (ViveBio Alpharetta, GA) for transport to the University of Georgia for further analyses.

### Cell surface staining and flow cytometry analysis

Circulating levels of T regulatory cells (Tregs) were determined at each time point for *S*. *mansoni* and STH positive individuals as well as a similar number of *S*. *mansoni* and STH negative controls utilizing a method described in detail previously [46]. Briefly, whole blood was stained with Alexa Fluor anti-CD3 clone UCHT1, PerCP anti-CD4 clone RPA-T4, and PE anti-CD25 clone BC96 (all from Biolegend, San Diego, CA). Samples were run on a FACS Calibur 4 color flow cytometer (BD Biosciences, San Jose, CA) and analyzed using FlowJo version 9 (Ashland, OR). Tregs were defined as the CD3^+^/CD4^+^/CD25^high^ population. Using fluorescence minus one (FMO) controls a gate was set to distinguish CD25^neg^ cells from CD25^med^ cells. CD4/CD25 dot plots were examined for all individuals in order to establish the threshold for the CD25^hi^ population.

### Cytokine production in response to vaccine antigens

Cytokine responses to vaccine antigens were determined at each time point for *S*. *mansoni* and STH positive individuals as well as a similar number of *S*. *mansoni* and STH negative controls. Under sterile conditions, whole blood was diluted 1:5 with cell culture media (RPMI 1640, 1% L-glutamine, 1% Penicillin-streptomycin) and cultured with phytohemagglutinin (PHA) (Sigma-Aldrich, St. Louis, MO) at a final concentration of 2.5 μg/ml, tetanus toxoid (TT) antigen (Mass Biologics, Boston MA) at a final concentration of 5 μg/ml, or hepatitis B surface antigen (HbSAg) (Reagant Proteins of Pfenex Dan Diego, CA) at a final concentration of 0.1 μg/ml. The cultures were allowed to incubate for 72 hours at 37C in 5% CO_2_ at which time culture supernatant fluids were collected and stored at -20C. IL-10, IL-5, and IFN-γ production in response to PHA, TT, and HbSAg were measured using Duoset ELISA kits (R&D systems, Minneapolis, MN) as recommended by the manufacturer.

### Measurement of antibody responses to hepatitis B proteins

Hepatitis B surface antigen antibody (anti-HBs) levels were quantitatively measured using Monalisa Anti-HBs EIA and Monalisa Anti-HBs calibrator kits (EIA) (BioRad, Redmond, WA) in baseline, 7 week and 8 month time point plasmas. Total antibody levels to hepatitis B nucleocapsid antigen (core) were qualitatively measured in baseline plasma samples using the Monalisa Anti-HBc Enzyme Immunoassay (EIA) (BioRad Redmond, WA). All assays were run as recommended by the manufacturer.

### Determining hepatitis B exposure status

We retrospectively allocated participants to the following categories. Previous exposure and recovery from hepatitis B infection or previous immunization with hepatitis B vaccine was defined as a baseline anti-HBs level above 10 mIU/ml [47]. Prior immunization would be very unlikely due to the relative expense and general lack of vaccine availability for adults in western Kenya. Potential hepatitis B chronic infection was defined as baseline antibody positive to hepatitis B core antigen, anti-HBs titers below 10 mIU/ml and failure to respond to the vaccine series [48,49] (levels never rose above 10 mIU/ml). Initial susceptibility to hepatitis B infection was defined as those individuals who at baseline were antibody negative to core antigen, had anti-HBs titers below 10 mIU/ml, and developed anti-HBS levels above 10 mIU/ml after vaccination [47]. Finally, persons with false positive core antibody responses were defined as individuals with baseline antibodies against core antigen, anti-HBs levels below 10mIU/ml, and the development of anti-HBs responses above 10 mIU/ml after vaccination [50–53].

### Measuring antibody responses to tetanus toxoid vaccine

Antibodies to TT were quantitatively measured using a commercially available tetanus toxoid IgG ELISA (Genway Platinum, San Diego, CA). Antibodies to TT were measured in baseline, 6 week and 8 month time point plasmas.

### Statistical methods

Data was entered into Microsoft Access 2010 databases. Individual datasets were generated using IBM SPSS version 23. GraphPad Prism version 5.04 for windows (Graphpad Software, San Diego, CA) was used for statistical analyses as well as for preparing graphs. The non-parametric Mann-Whitney test was used to compare anti-HbS levels, anti-TT levels, cytokine levels in response to vaccine antigen stimulation of whole blood, and to compare circulating CD3^+^CD4^+^CD25^high^ T-regulatory cells between controls and *S*. *mansoni* positive individuals at each of the time points- baseline, week 6 and month 8. To determine differences in CD3^+^CD4^+^CD25^high^ T-regulatory cell levels within group and over 3 time points a Friedman test followed by Dunn’s multiple comparison test was used. A chi square test was utilized to compare differences between the proportions of control versus *S*. *mansoni* positive individuals regarding social economic status (SES), water contact, hepatitis B virus exposure status before vaccination, and hepatitis B vaccine responder status after the 2^nd^ vaccine dose. A Fisher’s exact test was utilized to determine differences in the proportion of controls versus *S*. *mansoni* positive individuals maintaining TT antibody levels above 1 IU/ml at 6 weeks after boost and 8 months after boost.

## Results

### Demographic characteristics of study participants

**Fig 1:**
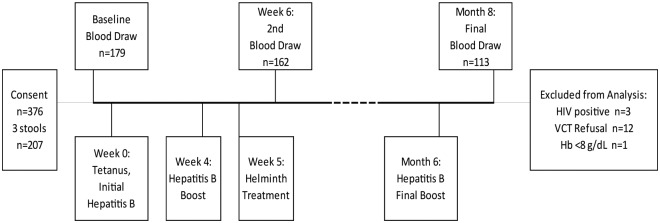
Study timeline showing study design and participant loss to follow-up. Students and staff were consented, enrolled into the study and required to submit 3 stool samples, receive hepatitis B vaccine series and tetanus toxoid boost, submit to blood draws, and attend a Volunteer Counseling & Testing clinic. A total of 376 individuals signed consent forms with 146 individuals giving at least 2 blood samples thus meeting the study requirements for inclusion in analyses.

This study took place in western Kenya from July 2013 until January 2015 at Kisumu Polytechnic College located in the center of Kisumu. The study location was selected because the staff and enrolled students were primarily from western Kenya, an area endemic for schistosomiasis so they were potentially infected with the parasite as children or adolescents. However, as the staff and students were living at or near the college in an urban setting, they were unlikely to be infected or re-infected with schistosomes after treatment during the 8 month follow-up period. The study timeline and number of persons lost to follow up is outlined in Fig 1. A total of 376 individuals signed consent forms and of those, 179 individuals gave a baseline blood sample. From this group, 162 (90.5%) study participants received the tetanus booster, at least 2 of the 3 doses of the hepatitis B vaccine series and gave a follow-up blood sample 2 weeks after the 2^nd^ hepatitis B vaccine dose. Twelve individuals were excluded from analysis for failing to show a VCT card by the end of the study period (proof they knew their HIV status) and 3 study participants (2%) were HIV-positive and excluded from analysis. One participant with a Hb level below 8 mg/dL at baseline was excluded from the study (Fig 1). Thus, data from a total of 146 individuals who gave at least 2 blood samples and met the study requirements were available to contribute to the final analyses.

### Parasitologic results

Of the 146 individuals available for analysis (summarized in Table 1) 53% were female and the median age was 21 years with a range of 18-57years. Malaria prevalence by smear positivity was 3% at baseline and remained below 3% throughout the study. Median hemoglobin (Hb) levels for participants were 13 mg/dL or above at each of the follow-ups. A total of 38 individuals (26%) were *S*. *mansoni* egg positive, at the time of their initial immunizations, as determined by Kato-Katz stool assay done on three consecutively collected stools, with two Kato-Katz slides per stool. The average arithmetic mean egg burden for those infected was 159.5 eggs per gram (EPG) of stool. Of the 38 individuals who were stool positive for *S*. *mansoni* 4 were also stool positive for *Trichuris trichiura* and 1 was stool positive for hookworm. In addition, 9 individuals who were *S*. *mansoni* egg negative were egg positive by Kato-Katz for a soil transmitted helminth (STH) infection with 4 hookworm positive, 2 *T*. *trichiura* positive, 2 *Ascaris lumbricoides* positive, and 1 both *A*. *lumbricoides* and *T*. *trichiura* positive. Finally, 99 individuals were egg negative for *S*. *mansoni* and STHs by Kato-Katz and made up the control, uninfected group. Age, sex, malaria, HIV, Hb levels and SES, as determined by the SES/water questionnaire (S2 Fig), did not differ significantly between the egg negative uninfected controls (controls) and the schistosomiasis egg positive group (Sm+). However, water contact did differ significantly (Chi square p < 0.001) between the 2 groups with the Sm+ group being more likely to have worked, bathed, washed items, and or collected water from Lake Victoria while in their home village.

**Table 1:** Baseline characteristics of 146 participants who met all study requirements, completed at least baseline and first follow-up, as well as received tetanus boost and a minimum 2 of 3 hepatitis B doses.

		Egg-(Kato Katz) (n = 99)	*S mansoni* Egg+(Kato Katz)* (n = 38)	STH Egg+(Kato Katz) (n = 9)	Total (n = 146)
Sex	Males n (%)	40 (40)	25 (66)	3 (33)	68 (47)
	Females n (%)	59 (60)	13 (34)	6 (67)	78 (53)
Age (years)	Range	18–50	19–57	20–24	18–57
	Mean	22.2	23.9	21.1	22.6
	Median	21	22	20	21
Malaria	Positive n (%)	3 (3)	1 (3)	0	4 (3)
	Negative n (%)	94 (95)	37 (97)	9 (100)	140 (96)
	Missing n (%)	2 (2)	0	0	2 (1)
Hb (g/dL)	Range	8–18	9–19	9–16	8–19
	Mean	12.8	13.9	12.0	13.0
	Median	13.0	14.0	12.0	13.0

*Note that 5 participants that are Sm egg +ve are also +ve for STH

### Characterization of potential past exposure to hepatitis B virus at baseline

In sub-Saharan Africa hepatitis B virus is most commonly acquired during early childhood [54]. The prevalence of this virus in Kenya has been estimated to be greater than 8% [55], with hepatitis B vaccination of infants in Kenya becoming standard after 2001 [56]. All of our participants were born prior to this time and less than 2% of participants self-reported having received all or part of the hepatitis B vaccine series. Therefore, hepatitis B exposure status at baseline before vaccination was defined by looking first at baseline antibody responses to hepatitis B core and surface antigens and then evaluating how individuals responded to the vaccine series. A total of 145 individuals (98 controls, 38 Sm+, and 9 STH+) received at least 2 doses of hepatitis B vaccine during the study and provided baseline (before vaccination) and post-vaccination blood samples (2 weeks after the second hepatitis B dose, which was 1 week post-praziquantel treatment for Sm+ group) as shown in the study timeline (Fig 1). Of these, 104 completed the 3 dose vaccine series and provided a final blood sample at 8 months (2 months after final immunization). We allocated participants to the following categories as described in the methods section: a total of 36 individuals (25%) were assigned to the previous exposure and recovery from hepatitis B category; 8 individuals (5%) were assigned to the category of possible chronic hepatitis B infection and excluded from analysis; 85 individuals (59%) were considered unexposed and susceptible to hepatitis B virus at baseline and were included in the final analysis; and 16 individuals (11%) were considered false positives to core antigen and therefore unexposed and susceptible to hepatitis B infection. There were no significant differences in the proportion or number of control, Sm+, or STH+ individuals assigned to each of the hepatitis B exposure categories as determined by Chi square analysis (p = 0.4) (Table 2).

**Table 2:** Percentage of participants falling into the catagories for hepatitis B virus exposure.

	Unexposed to Hepatitis B virus; Vaccine Responder	Unexposed to Hepatitis B virus; False Positive to core antigen; Vaccine responder	Unexposed to Hepatitis B virus; Vaccine Non-responder	Chronic Hepatitis B infection	Recovered; Immune to Hepatitis B infection	Total
**Control Group n (%)**	50 (50)	10 (10)	6 (6)	5 (5)	27 (28)	98
***S*. *mansoni* Group n (%)**	23 (60)	6 (16)	0 (0)	3 (8)	6 (16)	38
**STH Group n (%)**	6 (67)	0 (0)	0 (0)	0 (0)	3 (33)	9
**Total**	79	16	6	8	36	145

### Measuring antibody responses to hepatitis B vaccination

**Fig 2:**
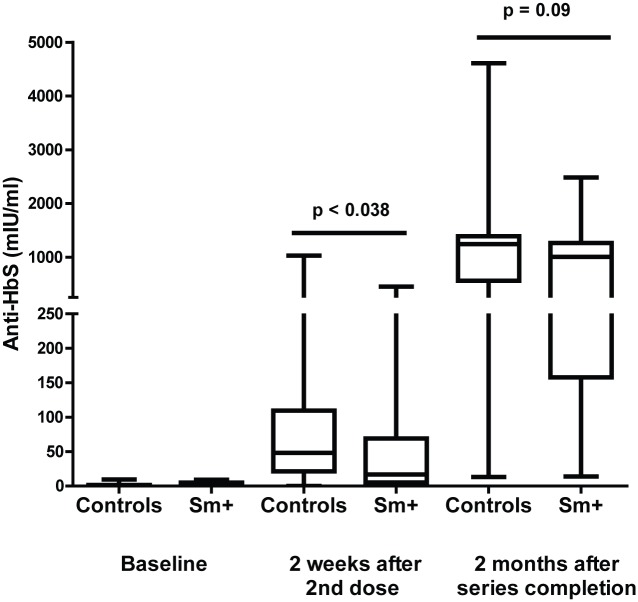
Schistosomiasis infection at the time of hepatitis B vaccination results in reduced antibody titers to hepatitis B surface antigen. Anti-HbS levels were measured by ELISA before vaccination, 2 weeks after 2^nd^ vaccine dose, and 2 months after vaccine series completion with individuals with schistosomiasis being treated for their infection 1 week following 2^nd^ dose of hepatitis B vaccine. Box plots show the median, lower 25^th^ percentile, and upper 75^th^ percentile anti-HbS levels at each time point. Statistical differences at each time point were determined using the Mann-Whitney test with a p < 0.05 considered statistically significant.

Of the 101 individuals deemed likely to be susceptible to hepatitis B virus infection at baseline, 6 individuals (6%) failed to achieve an anti-HBs response above 10 mIU/ml after receiving the full vaccination series and were classified as vaccine non-responders. This is within the expected range of non-response rate to hepatitis B vaccination of 4–10% [57,58] and these vaccine non-responders were excluded from the hepatitis B vaccine response analysis. The remaining 95 individuals (63% controls, 31% Sm+ and 6% STH+) all produced more than10 mIU/ml anti-HBs antibody levels after receiving the second or third dose of the vaccine series, indicating that they had achieved or exceeded the minimum level of protection. However, as blood concentration of anti-HBs antibodies have been shown to correlate with the maintenance of protective responses over time [59], we asked if study participants with *S*. *mansoni* infection at baseline (n = 29) or controls (n = 60) demonstrated differences in their median anti-HBs levels after 2 or 3 doses of vaccine. Median anti-HBs levels at 2 weeks after the second dose of hepatitis B vaccine were significantly lower (p = 0.038) in the Sm+ group as compared to the controls (Fig 2). By 2 months after the 3^rd^ dose of the vaccine (8 months from the initial dose and 7 months following treatment) the median response by those who had had schistosomiasis was still lower than those who were uninfected with the lower 25^th^ percentile being 157.8 mIU/ml compared to 560.5 mIU/ml for the controls. However, this difference was no longer statistically significant (p = 0.09) (Fig 2). We found that during the 6 months needed to complete the full hepatitis B immunization regimen approximately 30% of the participants were lost to follow-up or unavailable for 3rd dose of the hepatitis B vaccine. Therefore, we examined what percentage of participants receiving 2 doses did not produce more than 10 mIU/ml of specific antibody, the minimum level needed for protection. After the 2nd dose of vaccine 18% of the control group failed to mount a protective vaccine response (anti-HBs < 10 mIU/ml) as compared to 38% of the Sm+ group, and while 30% of the controls produce more than 100 mIU/ml of anti-HBs antibody (the highest category of protection), only 17% of the Sm+ group did so. While these differences failed to reach statistical significance (p = 0.10 by chi square analysis), they imply that individuals with schistosomiasis may be at a greater disadvantage than uninfected individuals if they do not to complete the full vaccine series. STH+ individuals (n = 6) antibody responses to hepatitis B vaccination are shown in S3 Fig. While the sample size for each was too small to run statistical analysis on (*A*. *lumbricoides* n = 2, hookworm n = 3, and *T*. *trichuria* n = 1), the median values of the STH+ group more closely resembled the controls than the Sm+ group (S3 Fig).

### Measuring antibody responses to tetanus toxoid booster vaccination

Vaccination against tetanus has been part of the extended program of immunizations in Kenya since 1980. It is also given to pregnant women as part of standard prenatal care [59] as well as individuals seeking care for injuries in a hospital settings. In our study, a total of 146 Sm+, STH+ and uninfected controls received the TT vaccine booster and gave a baseline and 7 week blood sample, with 113 participants giving a final blood sample at 8 months. Only 34% of participants recalled having ever received a tetanus booster vaccination, however all participants’ baseline TT titers were above 0.01 IU/ml (minimum level of protection) [44], indicating they had received at least one tetanus vaccination in the past. In order to account for the variation in participant tetanus vaccination histories as well as the impact that existing levels can have on recall responses [60,61], we analyzed participants according to three categories based on their baseline TT antibody titers: below 0.1 IU/ml; 0.1 to 1 IU/ml; and above 1 IU/ml [44]. For TT antibody titers below 0.1 IU/ml immediate immunization is recommended; 24 (16.5%) of all participants fell into this category. Sixty-three (43%) participants were categorized as needing immunization within 1 to 2 years (values of 0.1–1.0 IU/ml), and 59 (40.5%) individuals had levels greater than 1 IU/ml, for which immunization is recommended after 2 or more years.

**Fig 3:**
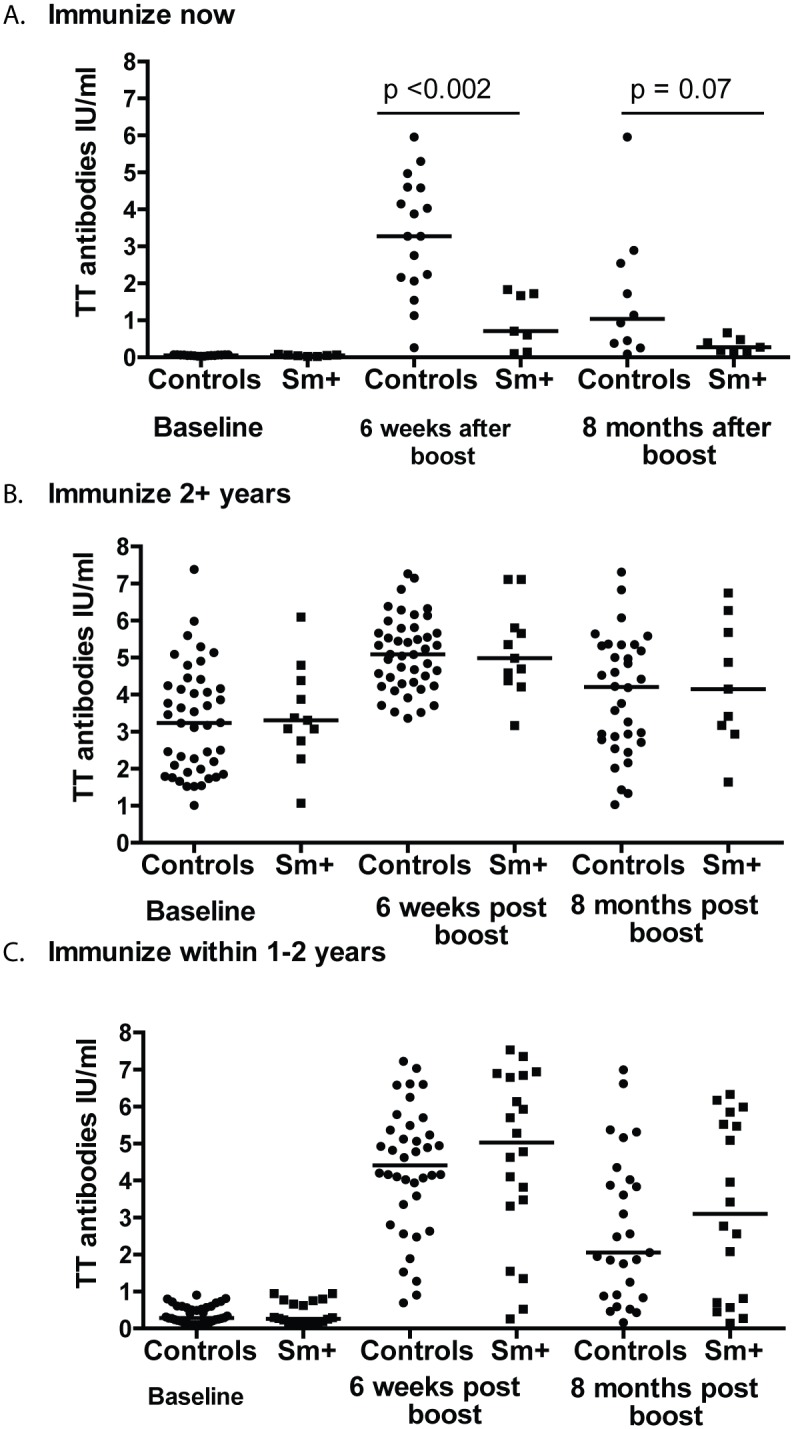
Individuals in need of immediate tetanus toxoid boost with a concomitant schistosomiasis infection do not respond as robustly to vaccination as compared to individuals without schistosomiasis at the time of vaccination. Participants were allocated to groups based on their need for a vaccine boost as determined by their baseline titers: Immunize now < 0.1mIU/ml (A.); immunize within 1–2 years 0.1 to 1.0 mIU/ml (B.); Immunize 2+ years > 1.0mIU/ml (C.). Anti-tetanus toxoid (TT) titers were measured by ELISA at baseline, 6 weeks after boost, and 8 months after boost. Circles represent study controls, individuals at baseline negative for *S*. *mansoni* and STHs, and squares represent those who were schistosomiasis positive at baseline. Bars represent median anti-TT levels at each time point and statistical differences at each time point were determined using the Mann-Whitney test with a p < 0.05 considered statistically significant.

When the anti-TT response of our participants in need of immediate boost were analyzed with respect to their *S*. *mansoni* infection status, the median antibody levels of the Sm+ group were significantly lower compared to the controls at 6 weeks (p < 0.002) after vaccination and relatively lower 8 months after receiving TT immunization (p = 0.07) (Fig 3A). For those categorized as needing a booster dose in 1–2 or more than 2 years, median antibody levels overlapped and did not differ between the Sm+ group and the control group (Fig 3B and 3C). As long term protection against tetanus is defined as having antibody concentrations above 1 IU/ml, we determined what percentage of participants produced this antibody level 6 weeks after booster vaccination and maintained that level for at least 8 months. At 6 weeks after vaccination, 97% of uninfected controls versus 84% of the schistosomiasis group had concentrations above 1 IU/ml. These differences were significant by Fishers exact test (p < 0.01). At 8 months after vaccination, 82% of uninfected controls maintained antibody levels above 1 IU/ml, compared to only 62% of the schistosomiasis group. These differences were significant by Fishers exact test (p < 0.03). Taken together, these data show that as a group, people with schistosomiasis are capable of responding to the tetanus toxoid boost but their antibody responses are not as robust as those of the control group and decline faster. This could leave them less protected over time and thus in need of more frequent TT immunization. STH+ individuals’ (n = 9) antibody responses to TT booster vaccination are shown in S4 Fig and again these were kept separate due to the small sample size. At baseline, all STH+ individuals were categorized as either needing immunization in 1 to 2 years or 2+ years. All STH+ participants responded to the booster vaccination with only 1 individual’s concentration dropping below 1 IU/ ml at 8 months (S4 Fig).

### Cytokine responses to hepatitis B surface antigen and TT

Robust responsiveness to hepatitis B vaccination has been previously associated with cytokine production in response to in vitro HBsAg stimulation [50]. In light of this finding, we evaluated IFN-γ, IL-5, and or IL-10 production in whole blood cultures in response to HbsAg stimulation before and after hepatitis B immunization and also analyzed if cytokine production was associated with antibody responses to the immunizations. We observed no differences in median levels of IFN-γ or IL-10 (Table 3) produced by the two groups in response to stimulation at any of the time points studied, nor did cytokine levels correlate with antibody responses. IL-5 was not produced by either group at any time point in response to HbSAg stimulation (Table 3).

**Table 3:** Median and Interquartile Range (IQR, 25^th^- 75^th^ percentile) of cytokine response (pg/ml) to 3 day whole blood culture with either tetanus toxoid or hepatitis B surface antigen.

		Controls		*S*. *mansoni*+	
		n	Median (IQR)	n	Median (IQR)
**Hepatitis B surface antigen stimulation**					
IL-5	Baseline	45	0 (0–0)	36	0 (0–0)
	6 weeks	42	0 (0–0.2)	36	1 (0–43)
	8 months	36	0 (0–0.7)	34	0 (0–2)
IL-10	Baseline	45	197 (68.5–406.5)	36	265 (87.7–501.8)
	6 weeks	42	380 (129.3–685.3)	36	494 (236.3–699.3)
	8 months	36	324 (137–571)	34	296.5 (129–701.5)
IFNγ	Baseline	45	0 (0–31)	36	0 (0–33.3)
	6 weeks	42	3.5 (0–57.5)	36	1 (0–18)
	8 months	36	3 (0–78.7)	34	0 (0–23)
**Tetanus toxoid stimulation**					
IL-5	Baseline	45	0 (0–2.5)	36	0 (0–7.5)
	6 weeks	42	1.0 (0–43)	36	9 (0–83.5)
	8 months	36	0 (0–15)	34	8 (0–47)
IL-10	Baseline	45	0 (0–0)	36	0 (0–0)
	6 weeks	42	0 (0–0)	36	0 (0–0)
	8 months	36	0 (0–3)	34	0 (0–0)
IFNγ	Baseline	45	0 (0–0)	36	0 (0–0)
	6 weeks	42	1.0 (0–38.75)	36	0 (0–8.5)
	8 months	36	0 (0–31.2)	34	0 (0–10.2)

**Fig 4:**
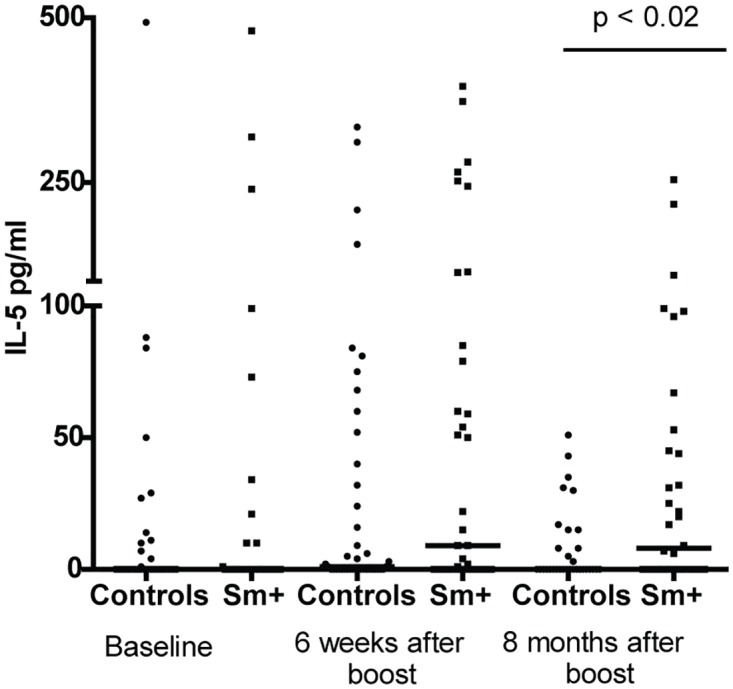
Individuals with schistosomiasis at the time of vaccination produced more IL-5 in response to in vitro tetanus toxoid stimulation compared to uninfected controls after vaccination. Whole blood cultures were stimulated in vitro with tetanus toxoid antigen for 72 hours and IL-5 was measured in the resulting supernatant fluids by ELISA at baseline, 6 weeks after boost and 8 months after boost. Circles represent study controls, individuals at baseline negative for *S*. *mansoni* and Soil Transmitted Helminths, and squares represent those schistosomiasis positive at baseline. Bars represent median IL-5 levels at each time point and statistical differences at each time point were determined using the Mann-Whitney test with a p < 0.05 considered significant.

We also examined cytokine responses to TT stimulation in whole blood cultures from individuals from the schistosomiasis and control groups at baseline, 6 weeks, and 8 months after immunization. We observed no differences in IFN-γ responses to TT antigen between participants with or without schistosomiasis at the time of TT immunization. Median IFN-γ levels were similar between the two groups as was the percentage of responders; i.e., proportion of individuals who produced IFN-γ in response to TT stimulation at baseline, 6 weeks and 8 months after vaccination (Table 3). However, there were differences in the IL-5 levels produced in response to TT stimulation. Median IL-5 levels were somewhat higher in the schistosomiasis group as compared to the uninfected controls at 6 weeks after vaccination, and significantly higher 8 months after immunization (p < 0.03) (Table 3, Fig 4). Furthermore, when IL-5 responses were examined in terms of individuals maintaining long-term antibody protection (above 1 IU/ml) at 8 months, we saw that individuals in the schistosomiasis group that had antibody titers above 1 IU/ml were more likely to produce IL-5 in response to TT stimulation. Fifteen out of 21 (71.4%) of long-term antibody producers mounted an IL-5 response compared to 4 out of 13 (30.8%) individuals whose anti-TT antibody titers had fallen below 1 IU/ml (p < 0.03). IL-10 was not produced in response to TT stimulation at any of the time points in either group (Table 3).

### Intensity of *S*. *mansoni* infection intensity does not influence antibody responses to vaccines in our cohort

Among the schistosomiasis group 24 (63%) were characterized as having a light infection (mean EPG less than 100), 11 (29%) had moderate infection (mean EPG 100–400) and only 3 (8%) heavy infection (mean EPG above 400) [62]. Previously, it has been reported that *S*. *mansoni* infection intensities influenced immune responses to TT vaccination [24]. However, in this study *S*. *mansoni* infection intensity, represented by EPG, failed to correlate with anti-HbS responses after 2 doses of vaccine and after the vaccine series was completed and median antibody levels did not differ significantly at each of the time points (S5A and S5B Fig). Infection intensities also failed to correlate with anti-TT titers at 6 weeks and 8 months after the booster vaccination was administered, with individuals with light, moderate or heavy infections being fairly evenly spread among those maintaining long-term protection at both 6 weeks and 8 months (S5C and S5D Fig).

### CD25hi T regulatory cells are elevated in schistosomiasis infected individuals but are not associated with antibody responses to immunizations

**Fig 5:**
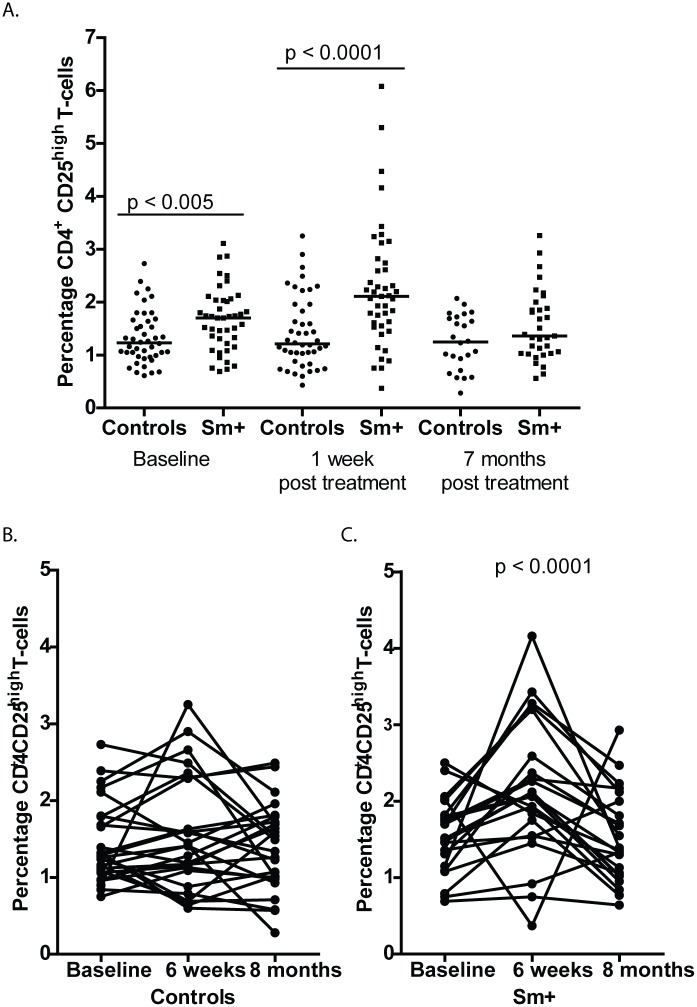
T regulatory cell (Treg) percentages (CD3^+^ CD4^+^ CD25^high^ cells) in peripheral blood are elevated in schistosomiasis infected individuals before praziquantel treatment and immediately following treatment. A. Whole blood was stained with anti-CD3-Alexa Fluor, anti-CD4-PercP and anti-CD25-PE and analyzed by flow cytometry at baseline, 1 week and 7 months following praziquantel treatment of schistosomiasis infected individuals and uninfected controls were followed at the same time points. CD25 expression was determined using a threshold based on florescence minus one (FMO) controls and used for all analyses with a CD25^high^ gate being set and used for all samples. Circles represent study controls, individuals at baseline negative for *S*. *mansoni* and Soil Transmitted Helminths, and squares represent schistosomiasis positive at baseline. Bars represent median Treg percentages at each time point and statistical differences at each time point were determined using the Mann-Whitney test with a p < 0.05 considered statistically significant. B. Treg percentages in controls at baseline, 7 weeks and 8 months did not change significantly over the study period as determined by the Friedman test followed by Dunn’s multiple comparisons. C. Treg percentages in Sm+ individuals increased significantly at 7 weeks, 1 week following PZQ treatment, as compared to baseline and 8 months as determined by the Friedman test followed by Dunn’s multiple comparisons.

CD3^+^ CD4^+^ CD25^hi^ T regulatory cells (Treg) are elevated in individuals with schistosomiasis [46], leading us to ask if those in our study had elevated levels of CD3^+^ CD4^+^ CD25^hi^ Treg, and if their Treg levels correlated with their antibody responses and for those who were Sm+ if their levels changed after treatment with praziquantel (PZQ). The *S*. *mansoni* group had significantly higher levels of CD3^+^ CD4^+^ CD25^hi^ Treg at baseline (before treatment) (p <0.005) and at 1 week after treatment (p < 0.0001) when compared to the uninfected control group, which remained steady throughout the study. By 7 months after treatment the elevated Treg levels in the *S*. *mansoni* group had then declined to levels seen in the uninfected control group (Fig 5A). For those members in the control and schistosomiasis groups for whom we had CD3^+^ CD4^+^ CD25^hi^ T-regulatory cells percentages at each time point we looked to see if those levels changed significantly between time points using the Freidman test followed by Dunn’s multiple comparison test. We saw no significant changes in Treg levels at each of the time points for the control group (Fig 5B). For the schistosomiasis group we saw significant differences in Treg levels with Treg levels increasing significantly between baseline and 1 week post praziquantel treatment and decreasing significantly 7 months following treatment (p < 0.0001 (Fig 5C). We also determined if an individual’s Treg levels correlated with their antibody responses to hepatitis B and TT immunizations. Treg individual levels did not correlate with individual antibody responses to either of the vaccines at any time points in either the schistosomiasis or control group, or with infection intensities in the schistosomiasis group (S6 and S7 Figs).

## Discussion

This study evaluated the immune responses of people with or without schistosomiasis mansoni upon primary immunization with the hepatitis B vaccine series and to a booster immunization with TT vaccine. Individuals in the study were in general good health. They did not have malaria and were not anemic. Those with helminth infections had generally quite light infections, i.e., low helminth egg excretion. Based on our findings, in this population it is clear that schistosome infections did not prevent production of vaccine-specific antibody responses. They did, however, alter the subsequent kinetics of vaccine-induced antibody responses, as seen in the significantly lower median anti-HBs levels after the second dose of hepatitis B vaccine and lower, but not significantly lower levels after the third dose of hepatitis B vaccine (Fig 2). This trend can also be seen in the proportion of individuals dropping below 1 IU/ml 8 months after TT boost, with 38% of schistosomiasis group falling below 1mIU/ml as compared to only 18% of controls.

The differences in the antibody levels achieved upon immunization with the hepatitis B series and TT are important as failure to maintain antibody levels could put the individual at risk over time. In regard to hepatitis B, vaccination studies in children immunized during infancy and early childhood have shown that the persistence of anti-HBs antibodies is influenced by initial antibody levels following vaccination, with higher initial levels correlating with the persistence of antibodies over time and mounting stronger anamnestic responses following booster vaccination [63–65]. One group suggested that individuals immunized as infants who failed to develop an anamnestic response following hepatitis B booster 15 years later showed a failure of immune memory and that this failure could put the now adolescents at risk if they were exposed to the virus [66,67]. However, as breakthrough infections following vaccination are infrequent and have not been shown to lead to chronic infection, subsequent booster doses for hepatitis B are not generally recommended except possibly for high risk groups [68]. Lower antibody levels to TT at the time of a TT boost and longer times between boosts lead to decreased responses to a booster vaccination [60,61,69]. Waning antibody responses have been linked to tetanus infections, particularly in the elderly [70]. Based on this established literature, we interpret our findings to indicate that although adults with generally low levels of *S*. *mansoni* infections respond adequately to hepatitis B vaccine and TT vaccine, they may be at risk of not achieving optimum or long last immune responses to these immunogens. Our study cohort did not include a sufficient number of individuals with STHs to comment adequately on the impact of STHs on these immunizations.

There are several important considerations of our data that cannot be addressed by our study. For example, while we assume that those with schistosomiasis acquired it during their childhood and have maintained it to their current age, we cannot know the duration of their schistosome infections. In addition, even with testing 3 consecutive stool specimens by 2 Kato-Katz slides each, we know this assay is relatively insensitive for detection of very low levels of *S*. *mansoni* infection [71] and thus some of our “uninfected control” individuals could actually have very light infections. In regard to this group, it is also possible that some of them have had schistosomiasis but lost their infections through the natural death of their worms since leaving their villages. With respect to hepatitis B, there are a substantial number of our study participants who appear to have been exposed to the pathogen and/or are currently infected. This is not surprising in this age group in western Kenya, as it is highly unlikely they would have been immunized as children but viral transmission in this area is relatively high [55,72]. Several studies of the effect of helminths on immune responses consider STHs, filarid and schistosome infections together. In our study we focused on a cohort we thought would have a reasonable number of schistosome-infected individuals who would not be likely to become re-infected during the follow-up period of 8 months, and in whom STH prevalence would likely be low. Studies on groups likely to have STHs would be of interest.

Perhaps the most important follow-on study based on our results would be to see if treatment of schistosomiasis with praziquantel would then render those treated more responsive to vaccination and if so, how long a waiting period after treatment would be needed before the immunizations would be most efficacious. Our data appear to indicate that such treatment prior to immunization would be beneficial, but the timing and actual impact of such schistosomiasis treatment remains to be determined.

Our studies were not designed to examine the mechanisms by which schistosomiasis might cause the effect we have observed on unrelated immunizations. We did however examine the levels of CD25+ T regulatory cells in our subjects. As previously reported, we found again [46,73] that people with schistosomiasis have elevated levels of these Tregs, and that these levels return to normal upon specific treatment with praziquantel. In addition, we demonstrated that immediately following praziquantel treatment their Treg levels increased and then dropped to the levels found in our uninfected control population. We did not, however, see any correlation between Treg levels and the resulting antibody levels upon the immunizations (S6 and S7 Figs). In examining other immune indicators and responses we noted that whole blood cultures from those individuals with schistosomiasis produced similar levels of IL-5, IFN-γ and IL-10 as compared to our uninfected controls after stimulation with HbSAg. In regards to TT stimulation, unlike what was previously reported [24], we did not see differences in IFN-γ levels in response to TT stimulation between our controls and schistosomiasis positive groups. However, in that study IFN-γ production was inversely related to infection intensity [24]. Our *S*. *mansoni* positive cohort had generally light infections and this may account for the differences seen between the studies. We did see differences in IL-5 production in response to whole blood stimulation with TT between control and *S*. *mansoni*-infected individuals. The schistosomiasis group’s median IL-5 levels in response to TT stimulation were higher than uninfected controls at 6 weeks and 8 months following boost. Surprisingly, we found that at 8 months IL-5 production in response to TT stimulation was associated with maintaining anti-TT levels above 1 IU/ml in individuals who were schistosomiasis positive at the time of their tetanus booster vaccination, suggesting that this Th2 bias response may have been beneficial to the maintenance of potentially protective antibody levels. Robust Th2 polarized memory responses have been seen against TT antigen in children immunized in infancy with the acellular diphtheria-tetanus-pertussis (DTaP) and then boosted in early childhood [74]. Interestingly, in that study 11 of the 19 children had a medical diagnosis of atopy or allergy. A study looking at adults also showed that atopy and asthma were also associated with the production Th2 cytokines, including IL-5 in response to TT stimulation [75], suggesting that in individuals biased towards IL-5 production to TT corresponds with strong responses to the vaccine.

In conclusion, we interpret our data to indicate that having even low levels of *S*. *mansoni* infection may influence the kinetics of vaccine induced antibody responses and that over time this could lower the continued maintenance of adequate immune responses against hepatitis B and TT vaccines. While this negative influence of schistosomiasis does not prevent responsiveness, it does lower the responses to a point where a person would be potentially at greater risk of acquiring hepatitis B and would need additional more frequent booster doses of TT to remain optimally protected against tetanus. This interpretation is in agreement with a study in semi-urban and rural Ugandan children, most of whom had *S*. *haematobium*, where the frequent boosting of the children during infancy and early childhood was required for the maintenance of robust TT antibody responses [34]. To fully address this point longer term studies are needed, but would be challenging to maintain. We believe these data indicate that it would be beneficial in terms of the efficacy of immunizations to treat people for their schistosomiasis prior to immunizations. Certainly in clinical studies of the efficacy of new candidate vaccines we propose that this approach would be important as the presence of schistosomiasis might increase the risk of a type 2 error. We hope that further studies will examine the potential effect of the timing of the recommended treatment for schistosomiasis to reverse its effect on vaccine responses. It is known that that praziquantel treatment initially augments some homologous anti-schistosome Th2-type responses [76,77]. However, due to our study design and the requirement for treatment of the participants infections we cannot currently address the critical question of whether for maximum effectiveness immunizations may need to be delayed for an as yet undetermined period of time after praziquantel treatment.

## Supporting Information

S1 Fig STROBE Checklist of study protocol. (DOCX)

S2 Fig Social economic status (SES) and water exposure questionnaire. Questionnaire was given at baseline to all consented participants to ascertain a participant’s SES and potential water exposures in their home villages that could have put them at risk of contracting *Schistosoma mansoni*.
(PDF)

S3 Fig Hepatitis B vaccine antibody responses for individuals with STH infection. Anti-HbS levels were measured by ELISA before vaccination, 2 weeks after 2^nd^ vaccine dose, and 2 months after vaccine series completion with individuals with STHs being treated for their infection 1 week following 2^nd^ dose of hepatitis B vaccine. Circles represent hookworm positive individuals, squares represent ascaris positive individuals and triangles represent trichuris positive individuals. Bars represent median anti-HbS levels at each time point.
(TIF)

S4 Fig Tetanus toxoid (TT) antibody responses for individuals with STH infection. Anti-tetanus toxoid levels were measured by ELISA before vaccination, 6 weeks after boost, 8 months after boost with individuals with STHs being treated for their infection 1 week following 2^nd^ dose of hepatitis B vaccine. Circles represent hookworm positive individuals, squares represent ascaris positive individuals, triangles represent trichuris positive individuals, and diamonds represent trichuris and ascaris co-infection. Bars represent median anti-TT levels at each time point.
(TIF)

S5 Fig *S*. *mansoni* infection intensity does not influence antibody responses to either hepatitis B or tetanus toxoid vaccines. Schistosomiasis positive individuals at baseline were grouped by their infection intensities light infection 1–99 mean EPG, moderate infection 100–399 mean EPG, and heavy infection 400 plus mean EPG. Bars represent median antibody levels at each time point. Red lines drawn at 1 IU/ml. Individuals falling below that line failed to achieve or maintain antibody levels necessary for long term protection.
(TIF)

S6 Fig CD3^+^ CD4^+^ CD25^hi^ T regulatory cell percentages did not correlate with individual antibody responses to hepatitis B vaccine. CD3^+^ CD4^+^ CD25^hi^ T regulatory cell percentages were measured by flow cytometry and anti-HbS levels were measured by ELISA at: (A) 2 weeks after the 2^nd^ vaccine dose; and (B) 2 months after the completion of the vaccine series. Individuals with schistosomiasis were treated for their infections 1 week following the 2^nd^ dose of hepatitis B vaccine. Linear regressions were performed on the data from both time points and neither was seen to be significant.
(TIF)

S7 Fig CD3^+^ CD4^+^ CD25^hi^ T regulatory cell percentages did not correlate with individual antibody responses tetanus toxoid (TT) boost. CD3^+^ CD4^+^ CD25^hi^ T regulatory cell percentages were measured by flow cytometry and anti-TT levels were measured by ELISA at: (A) baseline before boost; (B) 6 weeks after boost; and (C) 8 months after boost. Individuals with schistosomiasis were treated for their infections 5 weeks following the 2^nd^ TT boost. Linear regressions were performed on the data from all 3 time points and none were seen to be significant.
(TIF)
