# Supplementary material for: Schistosoma mansoni Infection Can Jeopardize the Duration of Protective Levels of Antibody Responses to Immunizations against Hepatitis B and Tetanus Toxoid
Source: PLoS Negl Trop Dis. 2016 Dec 7;10(12):e0005180. doi: 10.1371/journal.pntd.0005180 (PMC5142771; doi:10.1371/journal.pntd.0005180)
Supplement: S2 Fig — Questionnaire was given at baseline to all consented participants to ascertain a participant’s SES and potential water exposures in their home villages that could have put them at risk of contracting Schistosoma mansoni. (PDF) [file pntd.0005180.s002.pdf]

SES and water exposure questionnaire

[1] Participant ID: _____________

[2] Interview Date: __ __/ __ __ /__ __ __ __(Day/Month/Year)

[3] Interviewer's name: ____

[4] Participant's full name: ____

[5] What is your home district and county ____

a. Have you ever lived outside this area for longer than a year? ☐ Yes ☐ No

If yes, where? _______________ For how long? ______

[6] How long have you lived in Kisumu ______

A. If not from Kisumu when did you move to Kisumu? _________

[7] In your Family's house is there:

b. Electricity ☐ Yes ☐ No
c. Radio ☐ Yes ☐ No
d. Television ☐ Yes ☐ No
e. Refrigerator ☐ Yes ☐ No
f. Bicycle ☐ Yes ☐ No
g. Motorcycle ☐ Yes ☐ No
h. Car ☐ Yes ☐ No
i. Mobile phone ☐ Yes ☐ No
j. Domestic worker not related to household head ☐ Yes ☐ No

8) Do any members of your household work on their own or their family's agricultural land? ☐ es ☐ No

9) What is the principal source of drinking water in your family's household? *(Select one)*

k. Piped drinking water in residence
l. Piped drinking water in public tap
m. Piped drinking water in compound
n. Inside well drinking water
o. Public well
p. Rain for drinking water
q. River, canal or surface water for drinking
r. Other source of drinking water

10) What is the principal type of toilet facility used by the members of your household? *(Select one)*

s. Own flush toilet in residence
t. Shared flush toilet
u. Pit latrine
v. VIP latrine
w. Bush, field as latrine
x. Other type of latrine

11) What is the principal type of flooring in the dwelling where your family sleeps? *(Select one)*

y. Dirt, earth
z. Wood, planks
aa. Cement
bb. Tile flooring
cc. Other type of flooring

12) What is the principal type of roofing on the dwelling where your family sleeps? *(Select one)*

dd. Natural material roofing
ee. Corrugated iron
ff. Roofing tiles
gg. Other type of roofing

2) How often do you do your work by the lake (cleaning or selling fish, etc.)? (if never skip a-c)

☐ Never ☐ Sometimes ☐ Often

a. How long are you normally in contact with the water during this time?

☐ Briefly ☐ A while ☐ A long time

b. Which parts of your body are exposed to the water during this time?

☐ Arms ☐ Legs ☐ Head ☐ Whole body

c. At what time of day do you normally work by the lake?

☐ Morning ☐ Midday ☐ Afternoon ☐ Evening

3) How often do you bathe in the lake? (if never skip a-c)

☐ Never ☐ Sometimes ☐ Often

a. How long are you normally in contact with the water during this time?

☐ Briefly ☐ A while ☐ A long time

b. Which parts of your body are exposed to the water during this time?

☐ Arms ☐ Legs ☐ Head ☐ Whole body

c. At what time of day do you normally bathe in the lake?

☐ Morning ☐ Midday ☐ Afternoon ☐ Evening

4) How often do you wash things in the lake (clothes, household items including dishes)? (if never skip a-c)

☐ Never ☐ Sometimes ☐ Often

a. How long are you normally in contact with the water during this time?

☐ Briefly ☐ A while ☐ A long time

b. Which parts of your body are exposed to the water during this time?

☐ Arms ☐ Legs ☐ Head ☐ Whole body

c. At what time of day do you normally carry out this activity?

☐ Morning ☐ Midday ☐ Afternoon ☐ Evening

5) How often do you collect water from the lake? (if never skip a-c)

☐ Never ☐ Sometimes ☐ Often

a. How long are you normally in contact with the water during this time?

☐ Briefly ☐ A while ☐ A long time

b. Which parts of your body are exposed to the water during this time?

☐ Arms ☐ Legs ☐ Head ☐ Whole body

c. At what time of day do you normally carry out this activity?

☐ Morning ☐ Midday ☐ Afternoon ☐ Evening

6) Do you bathe, wash, or collect water from sources other than the lake more than once a week?

☐ Yes ☐ No

7) If yes, please check the source(s):

☐ Pond (continue with questions) ☐ River (continue questions) ☐ Well (no further questions)

How often do you do your work by the pond? (if never skip a-c)

☐ Never ☐ Sometimes ☐ Often

a. How long are you normally in contact with the water during this time?

☐ Briefly ☐ A while ☐ A long time

b. Which parts of your body are exposed to the water during this time?

☐ Arms ☐ Legs ☐ Head ☐ Whole body

c. At what time of day do you normally work in the pond?

☐ Morning ☐ Midday ☐ Afternoon ☐ Evening

8) How often do you bathe in water from the pond? (if never skip a-c)

☐ Never ☐ Sometimes ☐ Often

a. How long are you normally in contact with the water during this time?

☐ Briefly ☐ A while ☐ A long time

b. Which parts of your body are exposed to the water during this time?

☐ Arms ☐ Legs ☐ Head ☐ Whole body

c. At what time of day do you normally bathe in water from the pond?

☐ Morning ☐ Midday ☐ Afternoon ☐ Evening

9) How often do you wash things in the pond? (if never skip a-c)

☐ Never ☐ Sometimes ☐ Often

a. How long are you normally in contact with the water during this time?

☐ Briefly ☐ A while ☐ A long time

b. Which parts of your body are exposed to the water during this time?

☐ Arms ☐ Legs ☐ Head ☐ Whole body

c. At what time of day do you normally carry out this activity?

☐ Morning ☐ Midday ☐ Afternoon ☐ Evening

10) How often do you collect water from the pond? (if never skip a-c)

☐ Never ☐ Sometimes ☐ Often

a. How long are you normally in contact with the water during this time?

☐ Briefly ☐ A while ☐ A long time

b. Which parts of your body are exposed to the water during this time?

☐ Arms ☐ Legs ☐ Head ☐ Whole body

c. At what time of day do you normally carry out this activity?

☐ Morning ☐ Midday ☐ Afternoon ☐ Evening

16) How often do you do you do your work by the stream/river? (if never skip a-c)

☐ Never ☐ Sometimes ☐ Often

d. How long are you normally in contact with the water during this time?

☐ Briefly ☐ A while ☐ A long time

e. Which parts of your body are exposed to the water during this time?

☐ Arms ☐ Legs ☐ Head ☐ Whole body

f. At what time of day do you normally work in the stream/river?

☐ Morning ☐ Midday ☐ Afternoon ☐ Evening

11) How often do you bathe in the stream/river? (if never skip a-c)

☐ Never ☐ Sometimes ☐ Often

a. How long are you normally in contact with the water during this time?

☐ Briefly ☐ A while ☐ A long time

b. Which parts of your body are exposed to the water during this time?

☐ Arms ☐ Legs ☐ Head ☐ Whole body

c. At what time of day do you normally bathe in the stream/river?

☐ Morning ☐ Midday ☐ Afternoon ☐ Evening

12) How often do you wash things in the stream/river? (if never skip a-c)

☐ Never ☐ Sometimes ☐ Often

a. How long are you normally in contact with the water during this time?

☐ Briefly ☐ A while ☐ A long time

b. Which parts of your body are exposed to the water during this time?

☐ Arms ☐ Legs ☐ Head ☐ Whole body

c. At what time of day do you normally carry out this activity?

☐ Morning ☐ Midday ☐ Afternoon ☐ Evening

13) How often do you collect water from the stream/river? (if never skip a-c)

☐ Never ☐ Sometimes ☐ Often

a. How long are you normally in contact with the water during this time?

☐ Briefly ☐ A while ☐ A long time

b. Which parts of your body are exposed to the water during this time?

☐ Arms ☐ Legs ☐ Head ☐ Whole body

c. At what time of day do you normally carry out this activity?

☐ Morning ☐ Midday ☐ Afternoon ☐ Evening
