# Supplementary figures and images for: Schistosoma mansoni Infection Can Jeopardize the Duration of Protective Levels of Antibody Responses to Immunizations against Hepatitis B and Tetanus Toxoid
Source: PLoS Negl Trop Dis. 2016 Dec 7;10(12):e0005180. doi: 10.1371/journal.pntd.0005180 (PMC5142771; doi:10.1371/journal.pntd.0005180)

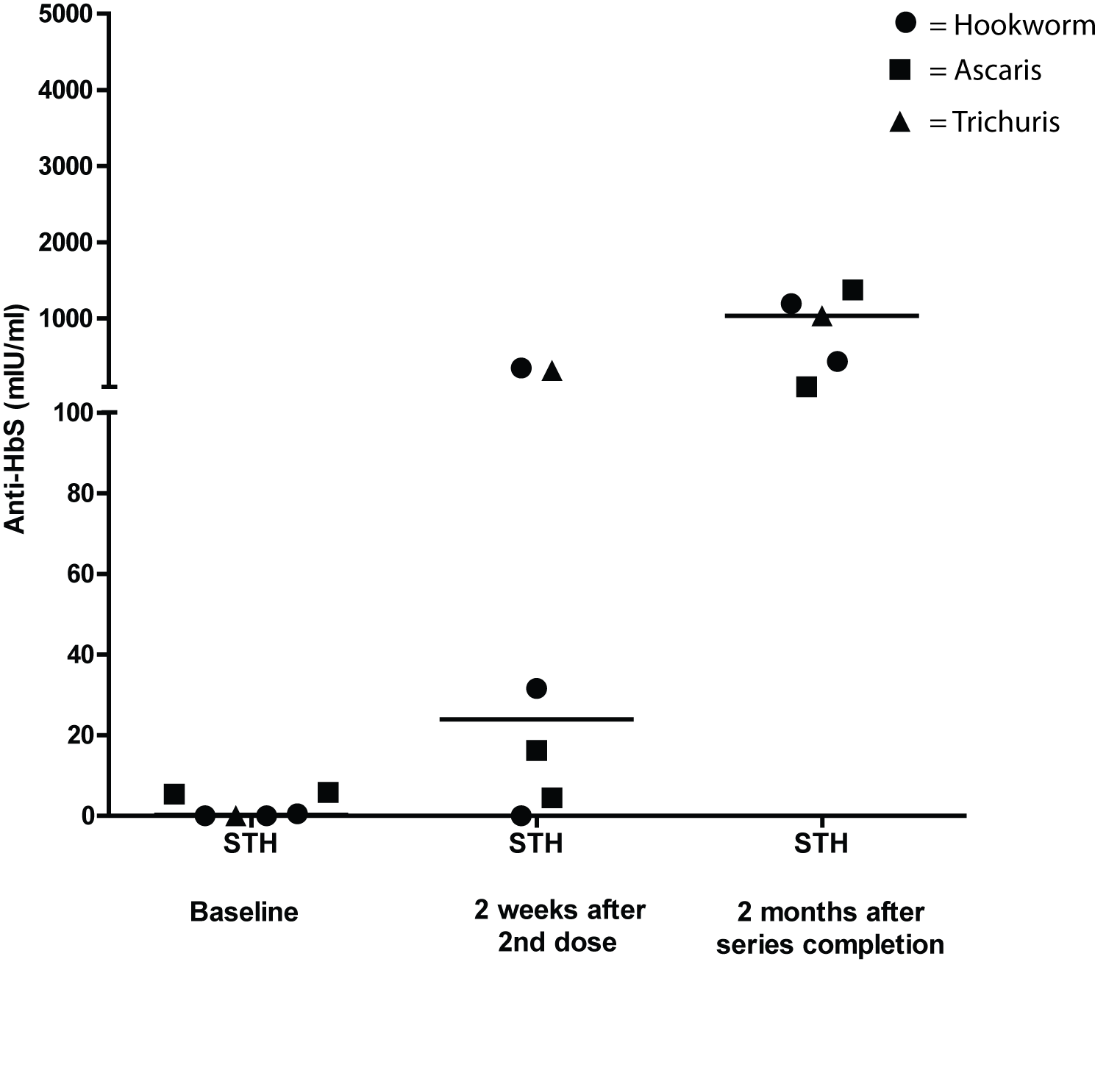

Supplement: S3 Fig — Anti-HbS levels were measured by ELISA before vaccination, 2 weeks after 2nd vaccine dose, and 2 months after vaccine series completion with individuals with STHs being treated for their infection 1 week following 2nd dose of hepatitis B vaccine. Circles represent hookworm positive individuals, squares represent ascaris positive individuals and triangles represent trichuris positive individuals. Bars represent median anti-HbS levels at each time point. (TIF) [file pntd.0005180.s003.tif]

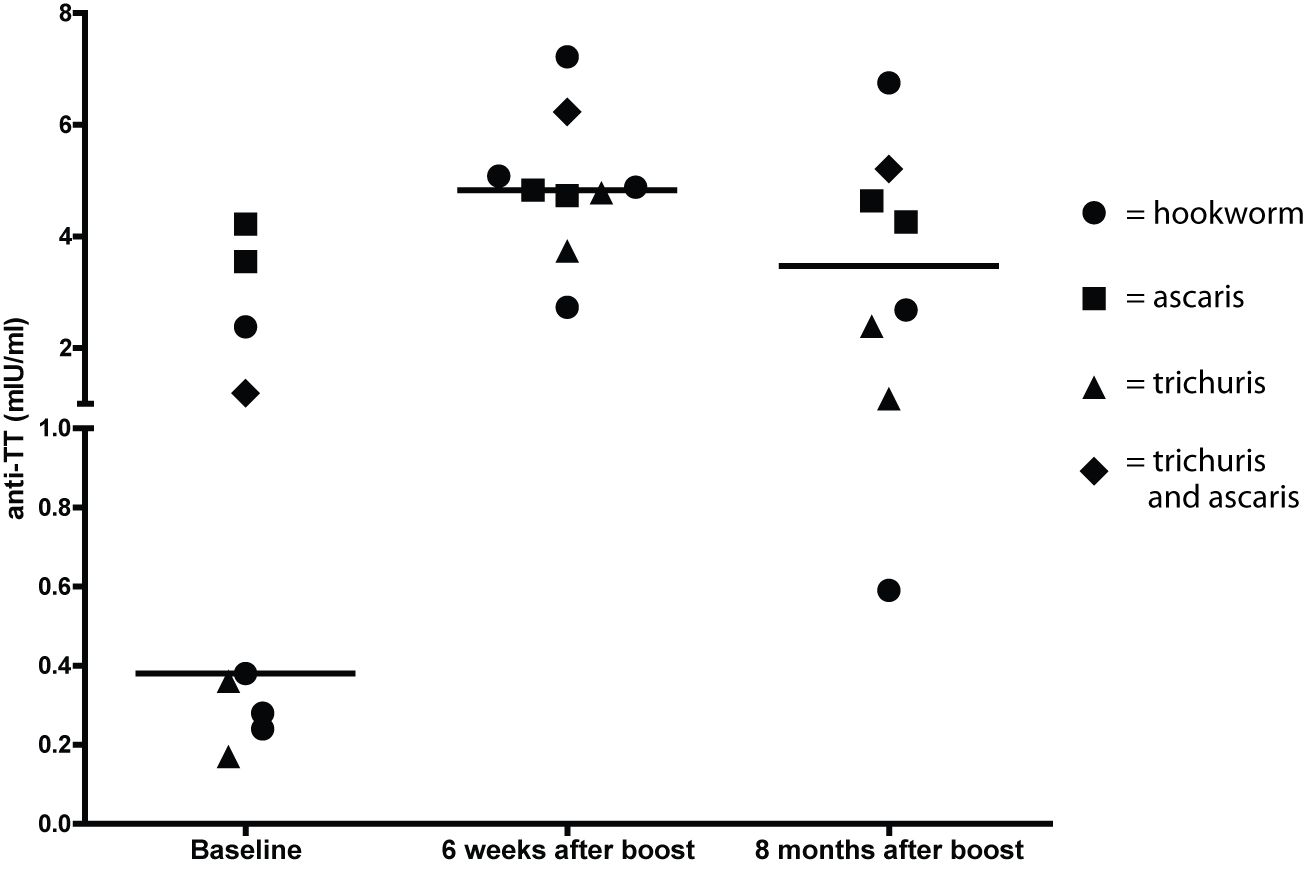

Supplement: S4 Fig — Anti-tetanus toxoid levels were measured by ELISA before vaccination, 6 weeks after boost, 8 months after boost with individuals with STHs being treated for their infection 1 week following 2nd dose of hepatitis B vaccine. Circles represent hookworm positive individuals, squares represent ascaris positive individuals, triangles represent trichuris positive individuals, and diamonds represent trichuris and ascaris co-infection. Bars represent median anti-TT levels at each time point. (TIF) [file pntd.0005180.s004.tif]

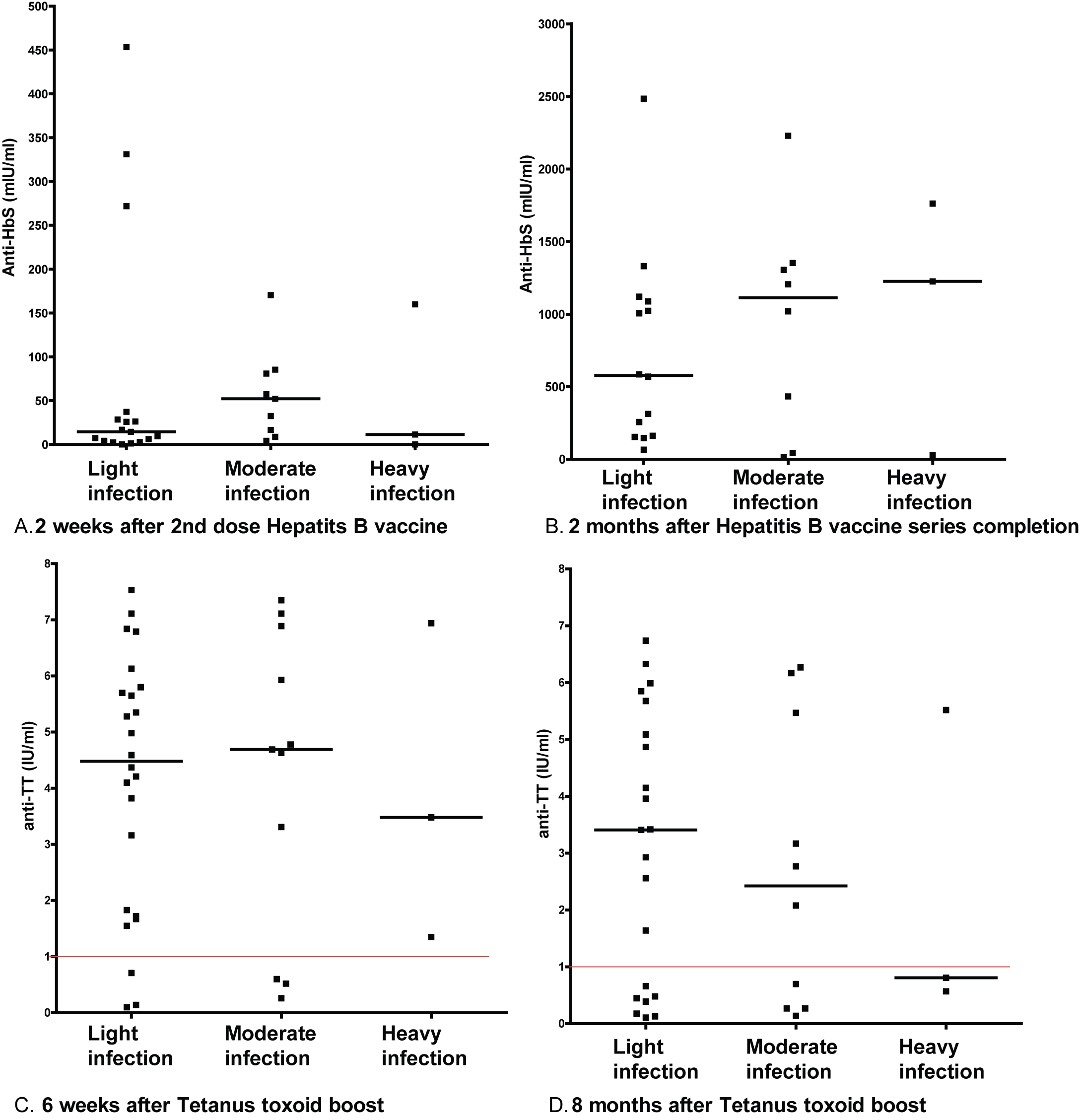

Supplement: S5 Fig — Schistosomiasis positive individuals at baseline were grouped by their infection intensities light infection 1–99 mean EPG, moderate infection 100–399 mean EPG, and heavy infection 400 plus mean EPG. Bars represent median antibody levels at each time point. Red lines drawn at 1 IU/ml. Individuals falling below that line failed to achieve or maintain antibody levels necessary for long term protection. (TIF) [file pntd.0005180.s005.tif]

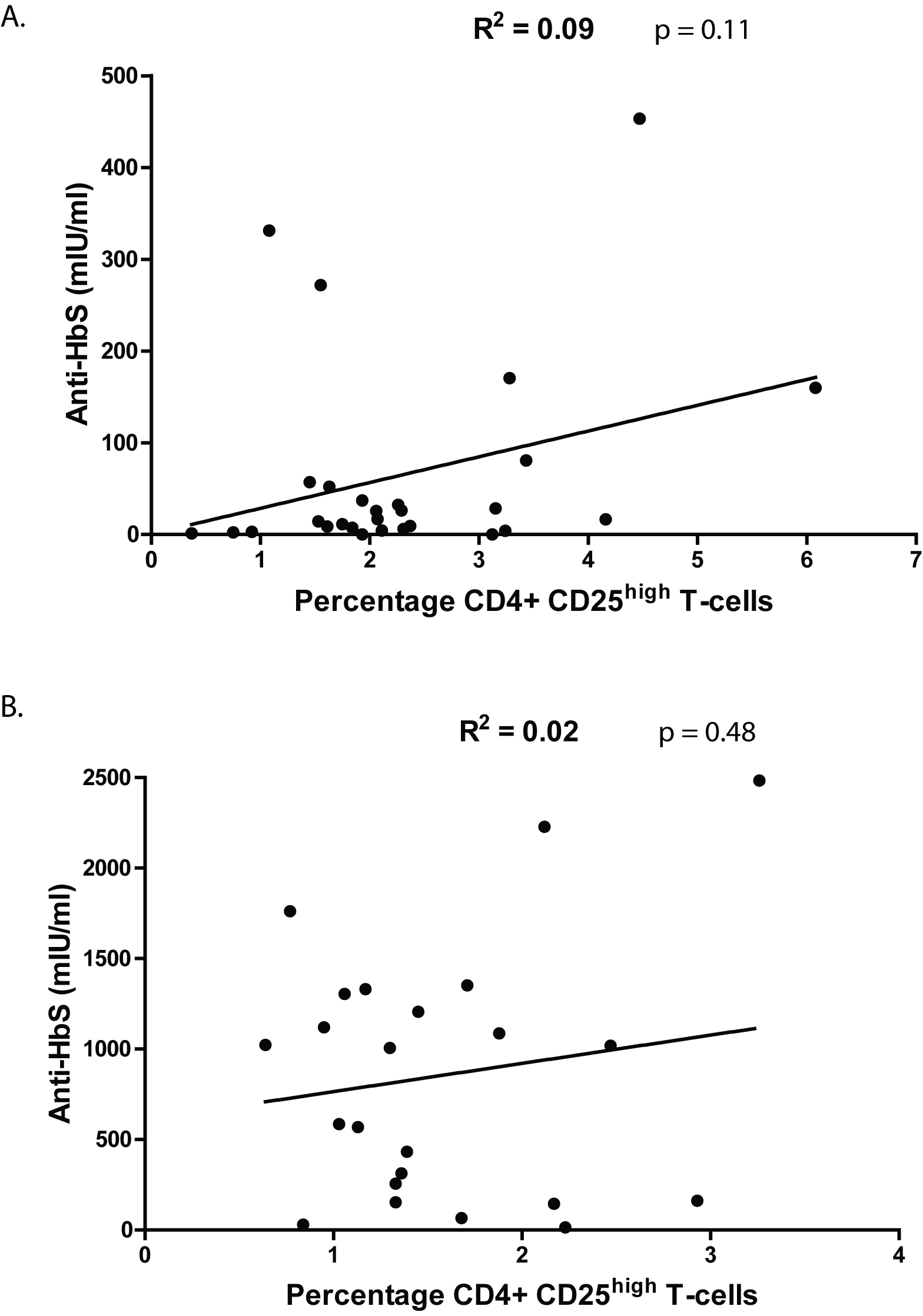

Supplement: S6 Fig — CD3+ CD4+ CD25hi T regulatory cell percentages were measured by flow cytometry and anti-HbS levels were measured by ELISA at: (A) 2 weeks after the 2nd vaccine dose; and (B) 2 months after the completion of the vaccine series. Individuals with schistosomiasis were treated for their infections 1 week following the 2nd dose of hepatitis B vaccine. Linear regressions were performed on the data from both time points and neither was seen to be significant. (TIF) [file pntd.0005180.s006.tif]

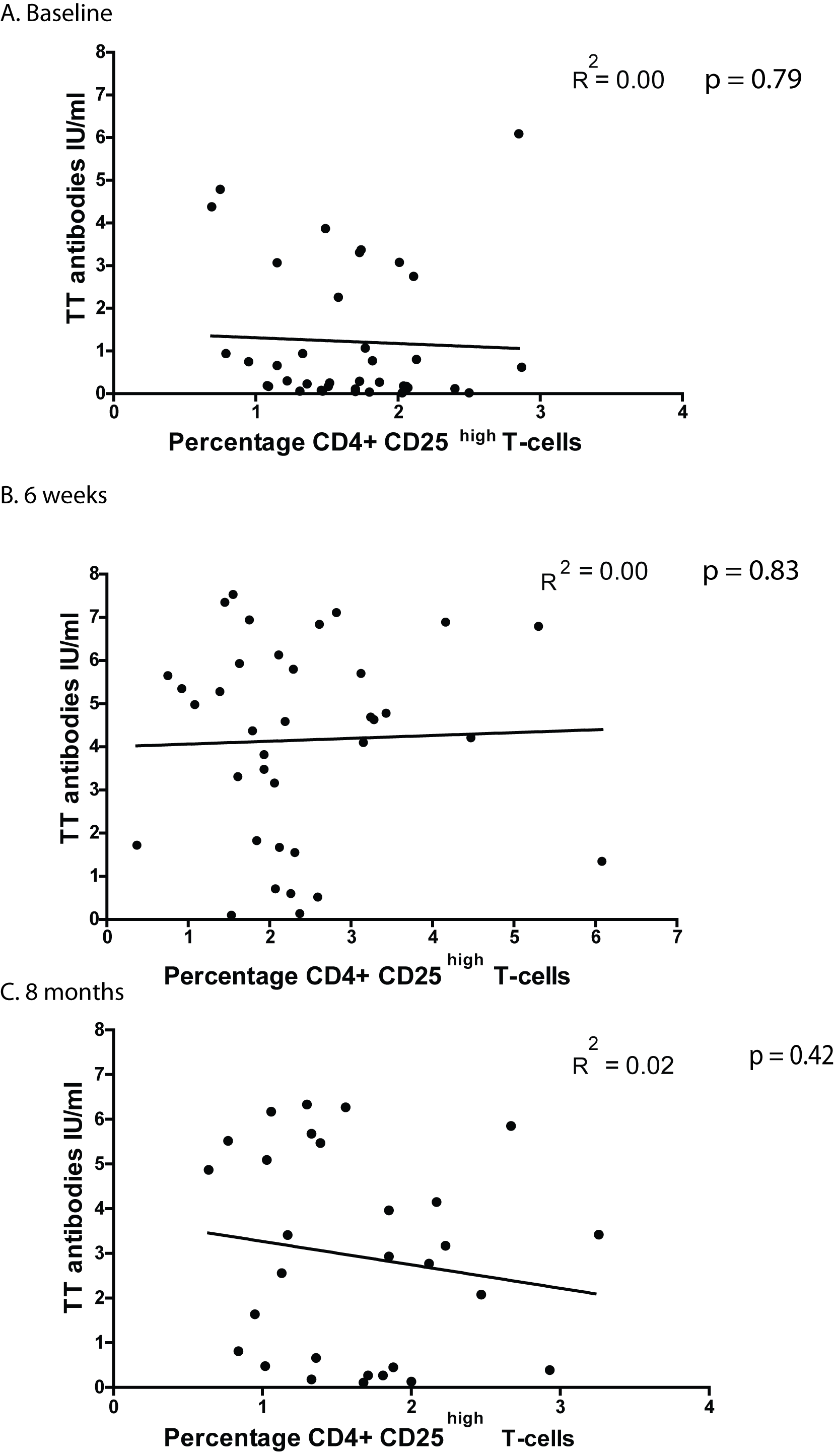

Supplement: S7 Fig — CD3+ CD4+ CD25hi T regulatory cell percentages were measured by flow cytometry and anti-TT levels were measured by ELISA at: (A) baseline before boost; (B) 6 weeks after boost; and (C) 8 months after boost. Individuals with schistosomiasis were treated for their infections 5 weeks following the 2nd TT boost. Linear regressions were performed on the data from all 3 time points and none were seen to be significant. (TIF) [file pntd.0005180.s007.tif]
